# O-Polysaccharide Plays a Major Role on the Virulence and Immunostimulatory Potential of *Aggregatibacter actinomycetemcomitans* During Periodontal Infection

**DOI:** 10.3389/fimmu.2020.591240

**Published:** 2020-10-30

**Authors:** Gustavo Monasterio, Francisca Castillo, Jessica Astorga, Anilei Hoare, Claudia Terraza-Aguirre, Emilio A. Cafferata, Eduardo J. Villablanca, Rolando Vernal

**Affiliations:** ^1^ Periodontal Biology Laboratory, Faculty of Dentistry, Universidad de Chile, Santiago, Chile; ^2^ Division of Immunology and Allergy, Department of Medicine, Karolinska Institutet and University Hospital, Stockholm, Sweden; ^3^ Center for Molecular Medicine, Stockholm, Sweden; ^4^ Oral Microbiology Laboratory, Faculty of Dentistry, Universidad de Chile, Santiago, Chile; ^5^ Institute for Regenerative Medicine and Biotherapies (IRMB), Université de Montpellier, Montpellier, France; ^6^ Department of Periodontology, School of Dentistry, Universidad Científica del Sur, Lima, Perú; ^7^ Department of Conservative Dentistry, Faculty of Dentistry, Universidad de Chile, Santiago, Chile

**Keywords:** periodontitis, *Aggregatibacter actinomycetemcomitans*, lipopolysaccharide, LPS, O-polysaccharide, O-PS, T-lymphocytes, bone resorption

## Abstract

*Aggregatibacter actinomycetemcomitans* is a Gram-negative oral bacterium with high immunostimulatory and pathogenic potential involved in the onset and progression of periodontitis, a chronic disease characterized by aberrant immune responses followed by tooth-supporting bone resorption, which eventually leads to tooth loss. While several studies have provided evidence related to the virulence factors of *A. actinomycetemcomitans* involved in the host cell death and immune evasion, such as its most studied primate-specific virulence factor, leukotoxin, the role of specific lipopolysaccharide (LPS) domains remain poorly understood. Here, we analyzed the role of the immunodominant domain of the LPS of *A. actinomycetemcomitans* termed O-polysaccharide (O-PS), which differentiates the distinct bacterial serotypes based on its antigenicity. To determine the role of the O-PS in the immunogenicity and virulence of *A. actinomycetemcomitans* during periodontitis, we analyzed the *in vivo* and *in vitro* effect of an O-PS-defective transposon mutant serotype *b* strain, characterized by the deletion of the *rmlC* gene encoding the α-L-rhamnose sugar biosynthetic enzyme. Induction of experimental periodontitis using the O-PS-defective *rmlC* mutant strain resulted in lower tooth-supporting bone resorption, infiltration of Th1, Th17, and Th22 lymphocytes, and expression of *Ahr*, *Il1b*, *Il17*, *Il23*, *Tlr4*, and RANKL (*Tnfsf11*) in the periodontal lesions as compared with the wild-type *A. actinomycetemcomitans* strain. In addition, the O-PS-defective *rmlC* mutant strain led to impaired activation of antigen-presenting cells, with less expression of the co-stimulatory molecules CD40 and CD80 in B lymphocytes and dendritic cells, and downregulated expression of *Tnfa* and *Il1b* in splenocytes. In conclusion, these data demonstrate that the O-PS from the serotype *b* of *A. actinomycetemcomitans* plays a key role in the capacity of the bacterium to prime oral innate and adaptive immune responses, by triggering the Th1 and Th17-driven tooth-supporting bone resorption during periodontitis.

## Introduction

*Aggregatibacter actinomycetemcomitans* is a small, non-motile, Gram-negative coccobacillus, resident in the oral cavity of humans and non-human primates, that preferentially colonizes the tissues that surround teeth (1). *A. actinomycetemcomitans* is strongly implicated in the pathogenesis of periodontitis, an inflammatory disease characterized by the disruption of the equilibrium among the periodontal microbiota and the host’s immune response, that subsequently leads to an increased local Th1 and Th17-type of response. Indeed, *A. actinomycetemcomitans* is considered a key player of the pathogenic consortium related to severe periodontitis, which causes accelerated periodontal tissue breakdown, in particular, the Th17-driven tooth-supporting bone resorption, the major pathological sign related with tooth loss (2, 3).

Alveolar bone resorption is the hallmark of periodontitis. During homeostatic conditions, the bone remodeling process is continuously maintained by the coupled action of bone-resorptive osteoclasts and bone-forming osteoblasts in a fine-tuned equilibrium regulated by a triad of molecules: the receptor activator of nuclear-factor κB ligand (RANKL), also known as tumor necrosis factor ligand superfamily member 11 (*Tnfsf11*), its specific receptor (RANK), and its soluble decoy osteoprotegerin (OPG) (4, 5). Indeed, RANKL is a key molecule involved in osteoclastogenesis and osteoclast-mediated bone resorption by stimulating the osteoclast progenitor differentiation and mature osteoclast activity (5). Otherwise, the immune response triggered against the dysbiotic subgingival microbiota during periodontitis disturbs the osteoblast/osteoclast equilibrium by dramatically increasing the RANKL local production and cellular sources, leading to pathological alveolar bone resorption (6, 7). Initial subversion of the host’s immunity by periodontal keystone pathogens and other oral bacteria promotes the accumulation of immunostimulatory pathobionts, such as *A. actinomycetemcomitans*, that finally contribute to immune-mediated alveolar bone loss (6–8).

Clinical evidence suggests a variable virulence potential among the distinct serotypes of *A. actinomycetemcomitans* (2, 9–11). Currently, seven *A. actinomycetemcomitans* serotypes are recognized based on the antigenicity of the O-polysaccharide (O-PS) immunodominant component of their lipopolysaccharide (LPS), being the serotypes *a*, *b*, and *c* the most frequently detected in humans (12–14). *A. actinomycetemcomitans* strains belonging to the serotype *b* are frequently isolated from subjects with severe periodontitis, while serotypes *a* and *c* are mostly isolated from milder periodontitis-affected patients and healthy individuals (2, 15–17). This variable pathological association has been related to the increased virulence of the serotype *b*, as we recently reported using an animal model of periodontitis, in which a higher lymphocyte Th1 and Th17-driven alveolar bone resorption was observed when the serotype *b* was used for periodontitis induction, as compared with the serotypes *a* or *c* of *A. actinomycetemcomitans* (18). Besides, *in vitro* studies have ratified the higher immunostimulatory potential of serotype *b* of *A. actinomycetemcomitans* on human antigen-presenting cells and T lymphocytes, as compared with the other serotypes (19–21), as well as its increased leukotoxin production (22). Indeed, most of the studies that analyze the higher virulence of serotype b of *A. actinomycetemcomitans* have focused on the production of leukotoxin, an exotoxin that selectively induces cell death in hematopoietic cells of human and non-human primate origin (23); however, the role of its O-PS has been scarcely studied.

The O-PS from *A. actinomycetemcomitans* serotype *b* is structurally distinct from the O-PS from the other serotypes. The O-PS from serotype *b* is composed of a disaccharide backbone of α-D-fucose (D-Fuc) and α-L-rhamnose (L-Rha), linked by a non-reducing β-D-N-acetyl-galactosamine (D-GalNAc) residue, and the O-PS from serotypes *a* and *c* consists of 6-deoxy- α-D-talose (α-D-Tal) and 6-deoxy-α-L-talose (α-L-Tal), respectively (24, 25). In the case of serotype *b*, the enzyme TDP-4-keto-6-deoxy-D-glucose 3,5-epimerase (RmlC) is required for the L-Rha synthesis and its assembling to the O-PS structure, and the genetic deletion of the *rmlC* gene encoding this enzyme completely abolish the production of the O-PS moiety, leading to a generation of an O-PS-defective *A. actinomycetemcomitans* strain (26). The *A. actinomycetemcomitans* strain lacking O-PS has shown altered bacterial adhesion and decreased leukotoxin secretion (26, 27); however, its pathogenic and immunostimulatory potential has not been analyzed yet.

In this study, we analyzed the role of the O-PS in the immunogenicity and virulence of *A. actinomycetemcomitans.* We took advantage of a murine model of periodontitis infected with the *rmlC* mutant strain, belonging to the serotype *b*. The deletion of the *rmlC* gene generated an O-PS-defective strain with reduced virulence, as demonstrated by the decreased tooth-supporting bone resorption in infected mice. This decreased alveolar bone loss was associated with downregulated levels of Th1 and Th17-related cytokines and less infiltration of Th1 and Th17 lymphocytes within the periodontal lesions. Besides, this O-PS-defective *A. actinomycetemcomitans* strain triggered a downregulated expression of *Tnfa* and *Il1b* in splenocytes and co-stimulatory molecules CD40 and CD80 in B lymphocytes and dendritic cells, in comparison to the wild-type and complemented strains. Collectively, these results demonstrate that the O-PS moiety from the serotype *b* of *A. actinomycetemcomitans* plays a key role in its immunostimulatory and virulent potential, affecting the maturation of antigen-presenting cells and the Th1 and Th17-driven alveolar bone resorption during periodontitis.

## Materials and Methods

### *A. actinomycetemcomitans* Strains

The *A. actinomycetemcomitans* mutant strains used in the current study are based on the nonfimbriated VT1169 strain belonging to the serotype *b* (all of them kindly provided by Dr. Keith Mintz, Department of Microbiology and Molecular Genetics, University of Vermont). In order to analyze the role of O-PS of *A. actinomycetemcomitans*, two mutant strains were used: the *rmlC* mutant strain, characterized by the absence of detectable O-PS, and the *waaL* mutant strain, characterized by impaired production of O-PS compared with the VT1169 wild-type strain, which was used for comparison (26). The transposon-generated *rmlC* mutant strain and the *waaL* mutant strain, generated by site-directed insertional mutagenesis, have been previously characterized (26). The *rmlC/rmlC^+^* and *waaL/waaL^+^* complemented strains, characterized by the restoration of the production of O-PS in a similar profile to the VT1169 wild-type strain, were used as controls (26). The *A. actinomycetemcomitans* strains were grown in 3% trypticase soy broth 0.6% yeast extract (TBSYE; Oxoid Ltd., England), supplemented with or without specific antibiotics, at 37˚C and under capnophilic conditions. The *rmlC* and *waaL* mutant strains were grown in the presence of 50 µg/ml spectinomycin, and the *rmlC/rmlC^+^* and *waaL/waaL^+^* complemented strains were grown in the presence of 1 µg/ml chloramphenicol. To obtain a reliable number of live bacteria with their whole antigenic potentiality for the periodontitis induction and cell stimulation, growth curves were made and bacteria were obtained at the exponential growth phase as previously described (28, 29). All the mutant and complemented *A. actinomycetemcomitans* strains used in the present study kept similar growth characteristics and colony morphology to the wild-type VT1169 strain.

### Lipopolysaccharide (LPS) Purification and Analysis

The LPS of the studied *A. actinomycetemcomitans* strains was purified using a modified hot phenol extraction protocol, based on a previously described protocol (30). LPS was visualized by Tricine-SDS-PAGE gel with 14% acrylamide/bis-acrylamide 46.5:3 solution, stained with a silver solution, as previously described (31). The gel was revealed in a solution containing 12.5 mg citric acid and 125 μL formaldehyde 37% in 250 mL of deionized water, photo-documented on a transilluminator, and analyzed using the Molecular Imaging Software “MI” 7.2 win (Bruker, Belgium).

### Animals

For periodontitis induction, eight-week-old wild-type BALB/c female mice were obtained at the estrus stage of the estrous cycle, to avoid the potential influence of sex hormones on bone metabolism. For *in vitro* assays, spleen cells were obtained from eight to sixteen-weeks-old C57BL/6 background mice. Animals were housed in separate cages and maintained under standard conditions: a 12:12 h light/dark cycle, lights on at 07:00 am, at 24°C ± 0.5°C, and 40% to 70% of relative humidity, with an air exchange rate of 15-room volumes/hour. Throughout the period of the study, animals had free access to sterile standard solid mice chow and water. Animals were handled according to the protocol approved by the Institutional Committee for Animal Care and Use from Universidad de Chile (Ethical permit #061601) and the Stockholm Regional Ethics Committee.

### Periodontal Infection

**Figure 1:**
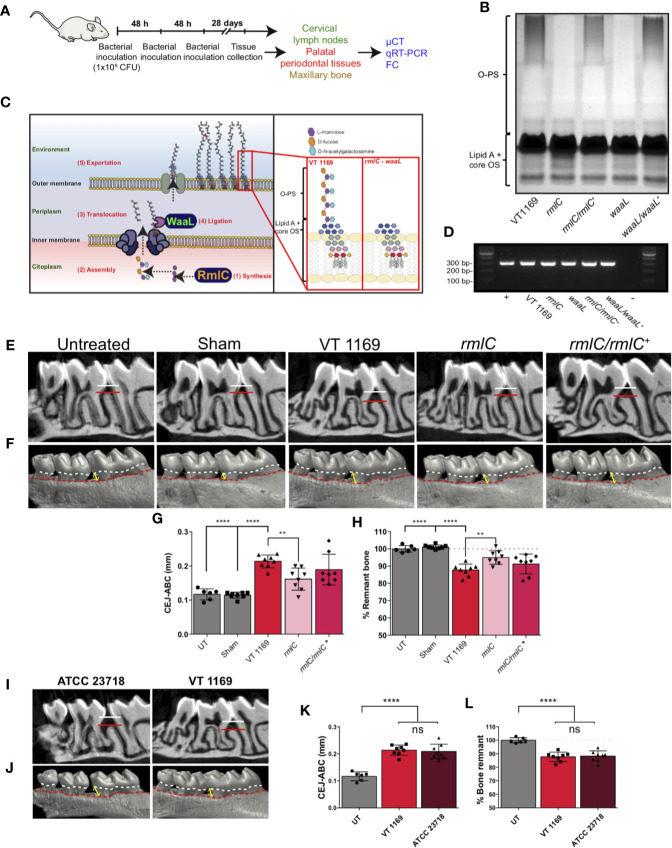
Lipopolysaccharide (LPS) analysis and alveolar bone loss triggered by the different strains of *A. actinomycetemcomitans*. **(A)** Diagram illustrating the protocol for the bacteria-induced periodontitis model. **(B)** LPS profiles obtained by SDS-PAGE analysis of the VT1169 wild-type strain and the *rmlC* and *waaL* mutant strains, as well as the *rmlC/rmlC^+^* and *waaL/waaL^+^* complemented strains. **(C)** Schematic view of the *A. actinomycetemcomitans* serotype *b* O-polysaccharide (O-PS) biosynthetic pathway, highlighting the modified enzymes and the O-PS profile obtained by Tang et al. (26) and used in the present study. **(D)** The 2% agarose gel of the amplification products obtained with specific primers for the strain VT1169, from PCR analysis of the V3-V6 variable region of the 16S rDNA. **(E)** Sagittal slices of the 2D-reconstruction by μCT showing the alveolar bone loss determined as the CEJ-ABC linear distance in the interdental area between the first and second molar (white-red lines) in mice infected with the *A. actinomycetemcomitans* VT1169, *rmlC*, or *rmlC/rmlC^+^* strains and the sham-infected and untreated controls. **(F)** Vestibular view of the 3D-reconstruction by μCT showing the alveolar bone loss, determined as the CEJ-ABC linear distance in the distal surface of the distal root of the first molar in the same experimental conditions described in **(E)**. Yellow double-arrow lines represent the CEJ-ABC linear distance (CEJ marked as a dashed white line and ABC marked as a dashed red line). **(G)** Quantification of the alveolar bone loss determined as the CEJ-ABC linear distance in the distal surface of the distal root of the first molar in mice infected with the *A. actinomycetemcomitans* VT1169, *rmlC*, or *rmlC/rmlC^+^* strains and the sham-infected and untreated controls (UT). **(H)** Quantification of the alveolar bone loss determined as the percentage of remnant alveolar bone in mice infected with the *A. actinomycetemcomitans* VT1169, *rmlC*, or *rmlC/rmlC^+^* strains and the sham-infected and UT controls, considering the average of remnant alveolar bone in UT mice as 100%. **(I)** Sagittal slices of the 2D-reconstruction by μCT showing the alveolar bone loss determined as the CEJ-ABC linear distance in the interdental area between the first and second molar (white-red lines) in mice infected with the *A. actinomycetemcomitans* VT1169 and ATCC 23718 strains. **(J)** Vestibular view of the 3D-reconstruction by μCT showing the alveolar bone loss, determined as the CEJ-ABC linear distance in the distal surface of the distal root of the first molar in the same experimental conditions described in **(I)**. Yellow double-arrow lines represent the CEJ-ABC linear distance (CEJ marked as a dashed white line and ABC marked as a dashed red line). **(K)** Quantification of the alveolar bone loss determined as the CEJ-ABC linear distance in the distal surface of the distal root of the first molar in mice infected with the *A. actinomycetemcomitans* VT1169 and ATCC 23718 strains and UT controls. **(L)** Quantification of the alveolar bone loss determined as the percentage of remnant alveolar bone in mice infected with the *A. actinomycetemcomitans* VT1169 and ATCC 23718 strains and UT controls, considering the average of remnant alveolar bone in UT mice as 100%. Mean ± SD, one-way ANOVA and Tukey post-hoc test, **p < 0.01, ****p < 0.0001. Error bars represent SEM in all panels. ABC, alveolar bone crest; CEJ, cement-enamel junction; CFU, colony-forming units; cLNs, cervical lymph nodes; FC, flow cytometry; ns, no significant; LPS, lipopolysaccharide; O-PS, O-polysaccharide; OS, oligosaccharide; PPTs, palatal periodontal tissues.

Periodontal infections were induced following a previously described protocol (18). Under ketamine/xylazine anesthesia, 2 μl of phosphate-buffered saline (PBS) cell suspension containing 1x10^9^ CFU/ml of each *A. actinomycetemcomitans* strain were microinjected using a 26s-gauge syringe (Hamilton, USA). A total of three microinjections were performed every 48 h into the palatal interproximal gingiva between the first and second molar of the right and left side of the maxilla (**Figure 1A**). Animals were randomly allocated in seven groups with three to four mice in each group: (a) VT1169 strain-infected group, (b) *rmlC* mutant strain-infected group, (c) *rmlC/rmlC^+^* complemented strain-infected group, (d) *waaL* mutant strain-infected group, (e) *waaL/waaL^+^* complemented strain-infected group, (f) sham-infected mice, which received PBS without bacteria, and (g) untreated animals. The mice were euthanized after 30 days by a single overdose of ketamine/xylazine anesthesia, and samples of maxillae, palatal periodontal tissues, and cervical lymph nodes were collected for further analysis. No changes were detected in the body-weight of mice throughout the study (**Supplementary Figure S1**).

### Quantification of Alveolar Bone Resorption

To quantify the extent of alveolar bone loss, the maxillae were scanned using a micro-computed tomography (μCT) equipment (SkyScan 1272; Bruker, Belgium), as previously described (18, 32). 3D-digitized images were generated using a modified cone-beam algorithm in a reconstruction software (Nrecon software, Bruker, Belgium). Alveolar bone loss was quantified using two methods: (a) by measuring the linear distance from the cement-enamel junction (CEJ) to the alveolar bone crest (ABC) and (b) by quantifying the percentage of remnant alveolar bone, by considering the average of untreated mice bone levels as 100%, as previously described (33, 34). In turn, the CEJ-ABC linear distance was quantified in (a) the interdental area comprised between the first and second molar and (b) the distal surface of the distal root of the first molar. All data were collected by a single observer (F.C.), who was masked to the conditions of the maxillae specimens.

### qRT-PCR From Palatal Periodontal Tissues

From the palatal periodontal tissues, total cytoplasmic RNA was purified, as previously described (18). After its quantification in a spectrophotometer (Synergy HT; Bio-Tek Instrument Inc., USA), the first-strand of cDNA was synthesized from 1 µg of total RNA using the SuperScript III kit (Invitrogen, USA), according to the manufacturer’s instructions. In order to quantify the mRNA expression levels for *Ahr*, *Ifng*, *Il6*, *Il10*, *Il17*, *Il22*, *Il23*, *Il1b*, *Tlr2*, *Tlr4*, and RANKL (*Tnfsf11*), 10 ng of cDNA were amplified using the appropriate primers (**Supplementary Table S1**) and the KAPA SYBR Fast qPCR reagent (KAPA Biosystems, USA), in a qPCR apparatus (StepOnePlus; Applied Biosystems, Singapore). Amplification reactions were conducted as follows: a first denaturation step of 95°C for 3 min, followed by 40 cycles of 95°C for 3 s and 60°C for 30 s. For detection of non-specific product formation and false-positive amplification, a final melting curve of 95°C for 15 s, 60°C for 1 min, and 95°C for 15 s was performed. Fold change of mRNA expression was calculated relative to the undisturbed 18S rRNA expression levels, using the 2^-ΔΔCt^ method followed by a log_2_-transformation.

### Single-Cell Suspension From Palatal Periodontal Tissues and Cervical Lymph Nodes

In order to obtain cells to perform the flow cytometric analysis, the palatal periodontal tissues and cervical lymph nodes that drain the periodontal tissues were processed, as previously described (32). For the recovery of palatal periodontal tissues, after accessing the oral cavity, whole maxillary blocks separated from the nasal cavity were dissected and immediately processed for enzymatic digestion. For this, specimens were immersed in 5 ml collagenase D/DNase (Sigma, USA) and incubated under constant agitation at 37°C for 1 hr. During the last 5 min of enzymatic digestion, specimens were incubated with 50 µl of 0.5 M EDTA (Sigma, USA) to stop the reaction. Then, in the presence of cold DNase media, the palatal periodontal tissues were removed from the maxillary bone, minced into 1 mm^3^ pieces, and mechanically mashed using a 70-μm cell-strainer, to obtain a single-cell suspension. After washing twice with cold DNase media, live cells were counted using an automated cell counter (Luna II; Logos Biosystems, South Korea). For the draining cervical lymph nodes, the cell suspensions were obtained through mechanic disruption of the three bilateral cervical lymph nodes that drain the periodontal tissues (mandibular, accessory mandibular, and superficial parotid), using a 70-μm cell-strainer in the presence of cold PBS.

### Flow Cytometry of Cells Obtained From Palatal Periodontal Tissues and Cervical Lymph Nodes

The presence of Tbet^+^ (Th1), RORγt^+^ (Th17), and AhR^+^ (Th22) lymphocytes within the periodontal lesions and cervical lymph nodes were analyzed by identifying the expression of their specific transcription factors using flow cytometry, as previously described (18, 32). The identified transcription factors were T-bet for Th1 cells, RORγt for Th17 cells, and AhR for Th22 cells. For this, the obtained cells were incubated for 15 min at 4°C with a Fc-blocking (CD16/32) antibody (eBioscience, Thermo Fisher Scientific, USA) prior to staining with fluorochrome-conjugated antibodies. For extracellular staining, the following lineage cocktail was used: CD45 (30-f11), CD4 (GK1.5), and CD3 (17A2). For intracellular staining, cells were incubated with a Fixation and Permeabilization buffer (Fix/Perm kit, eBioscience, Thermo Fisher Scientific, USA) for 30 min at 4°C followed by staining overnight at 4°C with the following antibodies: T-bet (4B10), RORγt (4G419), or AhR (4MEJJ). In addition, to analyze the immune cell compartment, the following cocktail was used: CD45.2 (104), CD19 (6D5), MHC-II (M5/114.15.1), CD3 (145-2c11), CD90 (53-2.1), CD11c (N418), CD11b (M1/70), Ly6C (HK1.4), Ly6G (1A8), and CD64 (54-5/7.1) (eBioscience, Thermo Fisher Scientific, USA; Biolegend, USA; and Abcam, England). Live/Dead Fixable viability dyes (eBioscience, Thermo Fisher Scientific, USA) were used to exclude dead cells. All the experiments were acquired using a FACS LSR Fortessa (BD Biosciences, USA) and analyzed with the FlowJo software (BD Biosciences, USA).

### Flow Cytometric Data Analysis by tSNE

T-distributed stochastic neighbor embedding (tSNE) analysis of 11-parameter flow cytometry data obtained from untreated mice was performed using the FlowJo software (BD Biosciences, USA). Samples were randomly downsampled to 10,000 events per sample, and analysis was run on equivalent numbers of events per sample. Following downsampling, 11-parameter samples were concatenated and visualized with tSNE (Barnes-Hut implementation) in FlowJo. The following parameters were tuned in preliminary experiments and then used as default values: iterations, 550; perplexity, 40; eta, 200; theta, 0.5. All parameters, except for lineage marker CD45 and DAPI live/dead marker, were included in the analysis.

### Total Splenocytes *In Vitro* Assays

Splenocytes were harvested from C57BL/6 background mice and maintained in IMDM medium (Gibco), supplemented with heat-inactivated 10% fetal calf serum (Sigma, USA), 100 U/ml penicillin, 100 μg/ml streptomycin, and 2% beta-mercaptoethanol (Gibco, Thermo Fisher Scientific, USA). Splenocytes (3x10^6^ cells/well) in 24-well plates were challenged with the *rmlC* or *waaL* mutant strains, the *rmlC/rmlC^+^* or *waaL/waaL^+^* complemented strains, or the VT1169 wild-type strain of *A. actinomycetemcomitans*, at a multiplicity of infection (MOI) 3 for 20 h for flow cytometric assays, or 8 h for qRT-PCR assays. Untreated cells or cells treated with 1 μg/ml of commercial *Escherichia coli*-derived LPS (Sigma, USA) were used as controls. The selected MOI was based on dose-response assays (**Supplementary Figure S2**).

### qRT-PCR From Induced Splenocytes

After 8 h of cell stimulation, splenocytes were washed and preserved in RNAlater at -80°C. Then, after cells were homogenized using a syringe, the total cytoplasmic RNA was isolated using the RNAeasy Mini Kit (Qiagen, Germany), and the first-strand cDNA was synthesized using the iScript RT Supermix (BioRad, USA), according to the manufacturer’s instructions. In order to quantify the mRNA expression levels for *Il1b*, *Il17*, *Il23*, and *Tnfa*, for the qRT-PCR analysis, the appropriate primers (**Supplementary Table S1**) and the iTaq Universal SYBR Green Supermix reagent (BioRad, USA) were used. Amplification reactions were conducted as follows: a first denaturation step of 95°C for 30 s, followed by 40 cycles of 95°C for 10 s, 60°C for 10 s, and 72˚C for 20 s. Fold change of mRNA expression was calculated relative to the *Hprt* expression levels, using the 2^-ΔΔCt^ method followed by a log_2_-transformation.

### Flow Cytometry of Stimulated Splenocytes

In order to analyze the surface expression of co-stimulatory molecules CD40 and CD80 in B lymphocytes, dendritic cells, and macrophages obtained from total splenocytes, the following antibodies were used: CD19 (6D5), MHC-II (M5/114.15.1), CD11c (N418), CD11b (M1/70), Ly6C (HK1.4), CD80 (16-10A1), and CD40 (HM40-3) (eBioscience, Thermo Fisher Scientific, USA; Biolegend, USA; and Abcam, England). Live/Dead Fixable viability dyes (eBioscience, USA) were used to exclude dead cells. All the experiments were acquired using a FACS LSR Fortessa X20 flow cytometer (BD Biosciences, USA) and analyzed with FlowJo software (BD Biosciences, USA).

### Statistical Analysis

Statistical analysis was performed using the GraphPad Prism v.4.01 software (GraphPad Software, Inc., USA). Heatmaps from qRT-PCR fold-change data were done in Excel v.16.39 software (Microsoft, USA). The normality of data distribution was established using the Kolmogorov-Smirnov test. When a parametric analysis was carried out, the statistical differences were determined using the ANOVA and the Tukey or Holm-Sidak post-hoc tests. When a nonparametric analysis was carried out, the statistical differences were determined using the Kruskal-Wallis and Dunn tests. A difference was considered significant when p < 0.05.

## Results

### Analysis of the LPS Purified From the Studied *A. actinomycetemcomitans* Mutant Strains

To confirm that the *A. actinomycetemcomitans* mutant strains used in this study were characterized by changes in the O-PS expression, we carried out the electrophoretic assays of their purified LPS. The silver-stained profile of the LPS obtained from both the *rmlC* and *waaL* mutant strains revealed the absence of the O-PS domain (**Figure 1B**), in accordance with the previously reported profiles (26) (**Figure 1C**). The *rmlC/rmlC^+^* and *waaL/waaL^+^* complemented strains displayed a restored LPS profile with the presence of a complete O-PS moiety (**Figure 1B**). All the bacterial strains used for periodontitis induction and cell challenge were positive for the *A. actinomycetemcomitans* serotype *b*, as demonstrated by the PCR analysis of the V3-V6 variable region of the 16S rDNA (**Figure 1D**).

### The O-PS-Defective *A. actinomycetemcomitans* Serotype *b* Mutant Strain Induces Less Alveolar Bone Resorption Than the Wild-Type Strain

To elucidate the pathogenic role of the O-PS moiety from the *A. actinomycetemcomitans* serotype *b*, we used a previously described experimental animal model of periodontitis (18), and the extent of the alveolar bone resorption was determined using μCT (**Figures 1E, F**). Mice infected with the VT1169 wild-type strain of *A. actinomycetemcomitans* exhibited greater alveolar bone loss as compared with both the untreated mice and sham-infected mice (**Figures 1E–H**). This higher alveolar bone loss was demonstrated by the increased CEJ-ABC linear distance in the interdental area between the first and second molar (**Figure 1E**), the enhanced CEJ-ABC linear distance in the distal surface of the distal root of the first molar (**Figures 1F, G**), and the decreased percentage of remnant alveolar bone (**Figure 1H**) at both sides of the maxilla. To corroborate that this higher pathogenic potential was attributed to the *A. actinomycetemcomitans* serotype *b* instead of only one strain belonging to this serotype, the alveolar bone resorption was also analyzed in mice infected with another serotype *b* strain, the reference strain *A. actinomycetemcomitans* ATCC 23718. Similar levels of alveolar bone loss were detected in mice infected with the ATCC 23718 or VT1169 strains, determined as the CEJ-ABC linear distance in the interdental area (**Figure 1I**), CEJ-ABC linear distance in the distal root of the first molar (**Figures 1J, K**), and percentage of remnant alveolar bone (**Figure 1L**). When the *A. actinomycetemcomitans* *rmlC* mutant strain was used to generate the periodontal infections, significantly lower alveolar bone resorption was observed as compared with the periodontal infections induced with the *A. actinomycetemcomitans* VT1169 wild-type strain (**Figures 1E, F**). In mice infected with the *rmlC* mutant strain, the quantitative analysis of the CEJ-ABC linear distance shown in **Figure 1F** revealed a significant reduction in the alveolar bone loss as compared with mice infected with the VT1169 strain, with an average ranging between 0.213 and 0.161 mm (**Figure 1G**). Besides, this reduction represented an average increase of 7.27% in the remnant alveolar bone in *rmlC* mutant strain-infected mice, as compared with the VT1169 strain-infected mice (**Figure 1H**). The recovering of the *rmlC* gene in the *rmlC/rmlC^+^* complemented strain of *A. actinomycetemcomitans* led to an increase in the alveolar bone loss in infected mice, reaching similar levels than those detected in the VT1169 strain-infected mice (**Figures 1E–H**). Collectively, these results demonstrated that the O-PS moiety component of the LPS is involved in the virulence of *A. actinomycetemcomitans* and particularly, in the tooth-supporting alveolar bone loss induced during experimental periodontitis.

### The O-PS-Defective *A. actinomycetemcomitans* Serotype *b* Mutant Strain Induces Decreased Th1, Th17, and Th22-Related Immune Responses Than the Wild-Type Strain

**Figure 2:**
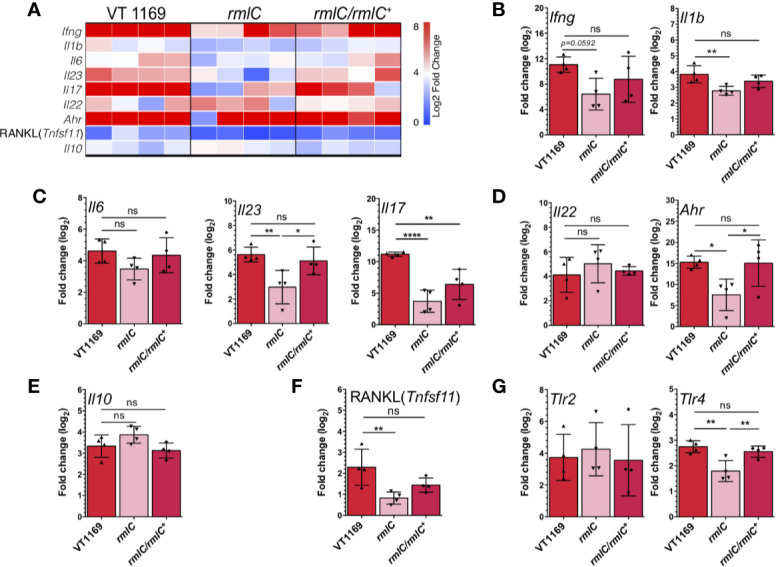
Th1/Th17/Th22-related cytokines, RANKL, and TLR expression within the periodontal tissues induced by the different strains of *A. actinomycetemcomitans*. **(A)** Heatmap of mRNA expression levels for Th1, Th17, Th22, and T regulatory-related cytokines and RANKL quantified by qRT-PCR. Bar plots showing relative expression of **(B)** Th1-related *Ifng* and *Il1b*, **(C)** Th17-related *Il6*, *Il17*, and *Il23*, **(D)** Th22-related *Il22* and *Ahr*, **(E)** T regulatory-related *Il10*, **(F)** RANKL (*Tnfsf11*), and **(G)** *Tlr2* and *Tlr4* mRNAs analyses in periodontal lesions infected with the *A. actinomycetemcomitans* VT1169, *rmlC*, or *rmlC/rmlC^+^* strains (n=4). The data were pooled from three independent experiments. For relative expression, the mRNA expression in untreated (UT) mice was considered as 0, as a reference for fold-change in expression. Mean ± SD, one-way ANOVA and Tukey post-hoc test, *p < 0.05, **p < 0.01, ****p < 0.0001, ns, non-significant. Error bars represent SEM in all panels. *Ahr*, aryl hydrocarbon receptor; *Il*, interleukin; *Ifng*, interferon-gamma; *Tlr*, toll-like receptor; RANKL (*Tnfsf11*), receptor activator of nuclear-factor κB ligand.

The immunogenic potential of the O-PS was assessed by analyzing the pattern of cytokines expressed in the periodontal lesions. For this, we determined the gene expression of the proinflammatory cytokines related with Th1 (*Ifng* and *Il1b*), Th17 (*Il6*, *Il23*, *and Il17*), Th22 (*Il22*), and T regulatory (*Il10*) lymphocyte responses in the palatal periodontal tissues by qRT-PCR (**Figure 2A**). Significantly, lower expression levels for *Ifng* and *Il1b* were detected in the periodontal lesions of mice infected with the *A. actinomycetemcomitans rmlC* mutant strain, as compared with those infected with the VT1169 wild-type strain (**Figure 2B**). Furthermore, when the *rmlC* mutant strain was used to induce the periodontal lesions, significantly lower expression levels of the Th17-related cytokines, *Il17* and *Il23*, were detected as compared with periodontal lesions induced with the VT1169 wild-type strain (**Figure 2C**). The *Il6* expression levels were also lower in the *rmlC* mutant strain-induced periodontal lesions as compared with the VT1169 strain-induced lesions; however, these differences were not statistically significant (**Figure 2C**). Similarly, the detected differences in the expression of the Th22-related cytokine *Il22* were not statistically significant (**Figure 2D**). As a complement of the analysis of the Th22-related immune response, the expression levels of *Ahr*, transcription factor master-switch gene associated with the Th22 lymphocyte differentiation and function, were also quantified. In mice infected with the *rmlC* mutant strain, the expression levels for *Ahr* were significantly lower than those detected in mice infected with the VT1169 wild-type strain (**Figure 2D**). No significant differences were detected in the transcript levels of the immunoregulatory cytokine *Il10* among the studied groups; however, there was a slight overexpression in the *rmlC*-induced periodontal lesions (**Figure 2E**). When the *rmlC/rmlC^+^* complemented strain was used to induce periodontal lesions, the expression levels for *Ifng*, *Il1b*, *Il6*, *Il10*, *Il22, Il23*, and *Ahr* reached similar levels to those detected in periodontal lesions induced with the VT1169 wild-type strain, except for *Il17*, whose expression levels remained lower (**Figures 2B–E**). Overall, these data demonstrated that the O-PS moiety of the LPS is implied in the immunogenicity of *A. actinomycetemcomitans*. In particular, the O-PS-defective mutant strain induced lesser Th1, Th17, and Th22-patterns of immune response in periodontal lesions than the *A. actinomycetemcomitans* wild-type strain, and these reduced levels are likely to be involved in the decreased alveolar bone resorption observed during the induced periodontitis.

### The O-PS-Defective *A. actinomycetemcomitans* Serotype *b* Mutant Strain Induces Less Expression of RANKL and TLR-4 Than the Wild-Type Strain

To confirm the association between the periodontal immune response and the observed changes in alveolar bone loss induced by the O-PS-defective *A. actinomycetemcomitans* mutant strain, we also determined the gene expression of the pro-osteolytic factor RANKL (*Tnfsf11*), the key factor involved the osteoclast differentiation and activation. In periodontal lesions induced with the *A. actinomycetemcomitans rmlC* mutant strain, the expression levels for RANKL (*Tnfsf11*) were significantly lower as compared with those induced with the VT1169 wild-type strain (**Figure 2F**). When the *rmlC/rmlC^+^* complemented strain was used for the periodontitis induction, the transcript levels of *Tnfsf11* did not reach the levels expressed in the VT1169 wild-type strain-induced periodontal lesions; however, these differences were not statistically significant (**Figure 2F**). In order to get insights into the potential pathways involved in the O-PS recognition, we also quantified the expression levels of two Toll-like receptors related to LPS signaling, TLR-2 and TLR-4. In the periodontal lesions induced with the *A. actinomycetemcomitans rmlC* mutant strain, significantly lower transcript levels of *Tlr4* were detected, as compared with those induced with the VT1169 wild-type strain (**Figure 2G**). No differences were detected for the *Tlr2* transcript levels. When the *rmlC/rmlC^+^* complemented strain was used for the periodontitis induction, the expression of *Tlr4* reached similar levels to the ones detected in the VT1169 wild-type strain-induced periodontitis lesions (**Figure 2G**). This result suggests that the effect in the *Tlr4* expression could be attributed to the absence of O-PS, due to deletion in *rmlC* gene.

### The O-PS-Defective *A. actinomycetemcomitans* Serotype *b* Mutant Strain Induces Less Detection of T-bet^+^, RORγt^+^, and AhR^+^ T Lymphocytes Than the Wild-Type Strain in the Periodontal Lesions

**Figure 3:**
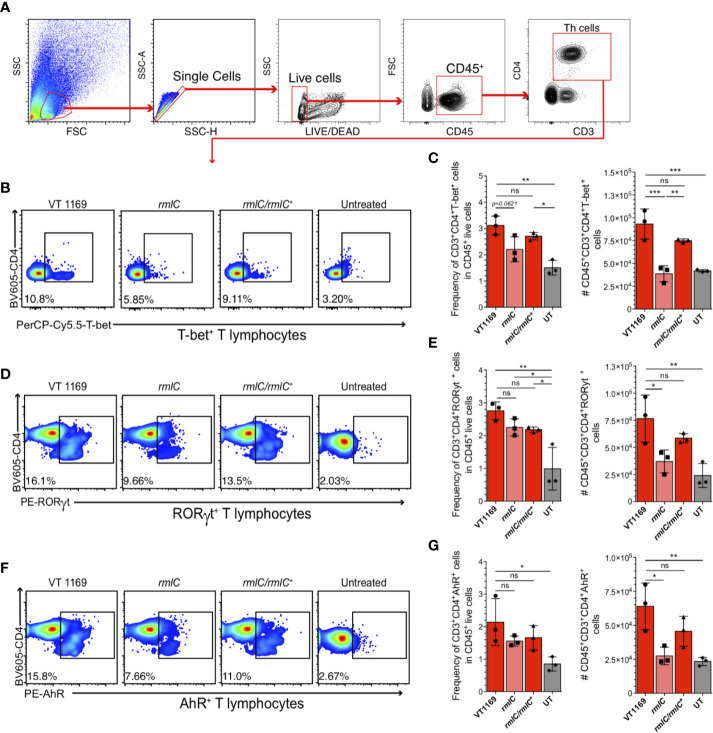
T-bet^+^, RORγt^+^, and AhR^+^ T lymphocytes detection within the periodontal tissues induced by the different strains of *A. actinomycetemcomitans*. Cells obtained from periodontal tissues were analyzed by flow cytometry. **(A)** Gating strategy used for lineage determination of single/live/CD45^+^CD3^+^CD4^+^ T lymphocytes. Flow cytometric quantification of **(B**, **C)** CD45^+^CD3^+^CD4^+^T-bet^+^ Th1 lymphocytes, **(D**, **E)** CD45^+^CD3^+^CD4^+^RORγt^+^ Th17 lymphocytes, and **(F**, **G)** CD45^+^CD3^+^CD4^+^AhR^+^ Th22 lymphocytes in periodontal lesions upon infection with the *A. actinomycetemcomitans* VT1169, *rmlC*, or *rmlC/rmlC^+^* strains, or the untreated (UT) controls. Plots are representative of three independent experiments (n=3). Mean ± SD, one-way ANOVA and Holm-Sidak post-hoc test, *p < 0.05, **p < 0.01, ***p < 0.001, ns, non-significant. Error bars represent SEM in all panels.

Since our data showed the decreased expression levels of the Th1, Th17, and Th22-related cytokines in the periodontal lesions induced with the *A. actinomycetemcomitans* serotype *b* strain lacking the O-PS domain, we proceeded to determine the frequency and absolute number of the T helper lymphocytes infiltrating these periodontal lesions (**Figure 3A**). Flow cytometry analysis revealed a higher frequency and absolute number of Th1 (CD45^+^CD3^+^CD4^+^T-bet^+^) (**Figures 3B, C**), Th17 (CD45^+^CD3^+^CD4^+^RORγt^+^) (**Figures 3D, E**), and Th22 (CD45^+^CD3^+^CD4^+^AhR^+^) (**Figures 3F, G**) lymphocytes in periodontal lesions of mice infected with the VT1169 wild-type strain compared with the periodontal tissues of untreated control mice. No differences were found in T-cell viability among the different experimental conditions, with an average of 95.81% viability of cells obtained from infected periodontal lesions (**Supplementary Figure S3**). Within periodontal lesions induced with the *rmlC* mutant strain, the T-bet^+^ T lymphocyte detection was significantly lower in frequency and absolute number, as compared with the VT1169 wild-type strain-induced periodontal lesions (**Figures 3B–C**). When the periodontal lesions were induced with the *rmlC/rmlC^+^* complemented strain, the frequency and absolute number of T-bet^+^ T lymphocytes increased, reaching levels similar to those detected in the periodontal lesions induced with the VT1169 wild-type strain (**Figures 3B–C**). Similarly, the absolute number of infiltrating RORγt^+^ T lymphocytes was significantly decreased by almost half, within the *rmlC* mutant strain-induced periodontal lesions as compared with the VT1169 wild-type strain-induced periodontal lesions (**Figures 3D–E**). No differences were detected in the frequency of RORγt^+^ T lymphocytes between the periodontal lesions induced with the *rmlC* mutant strain and *rmlC/rmlC^+^* complemented strain; however, when the absolute cell number was analyzed, a no-significant increment in the RORγt^+^ T lymphocyte detection was observed in the *rmlC/rmlC^+^* complemented strain-induced periodontal lesions (**Figure 3E**). Since it was recently suggested that Th22 lymphocytes could be associated with the RANKL-mediated alveolar bone loss in a mice model of *A. actinomycetemcomitans*-induced experimental periodontitis (32), we also compared the infiltration of AhR^+^ T lymphocytes within the periodontal lesions. No significant differences were detected in the frequency of AhR^+^ T lymphocytes between the *rmlC* mutant strain-induced and VT1169 wild-type strain-induced periodontal lesions (**Figures 3F, G**); however, a significantly lower AhR^+^ T lymphocyte number was quantified in periodontal lesions induced with the *rmlC* mutant strain as compared with those induced with the VT1169 wild-type strain (**Figures 3F, G**). When the *rmlC/rmlC^+^* complemented strain was used to induce periodontal lesions, the frequency and absolute number of Th22 reached similar levels to the ones detected in periodontal lesions induced with the VT1169 wild-type strain (**Figures 3F, G**). Taken together, these results allow us to suggest that the absence of the O-PS domain in the LPS from *A. actinomycetemcomitans* serotype *b* significantly affects its capacity to induce Th1, Th17, and Th22 lymphocyte infiltration in experimental periodontal lesions.

**Figure 4:**
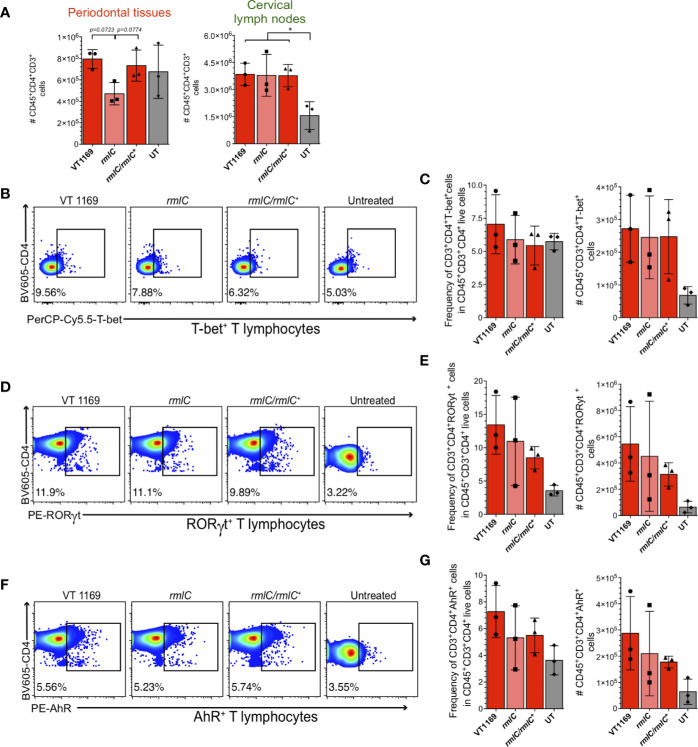
T-bet^+^, RORγt^+^, and AhR^+^ T lymphocytes detection within the cervical lymph nodes induced by the different strains of *A. actinomycetemcomitans*. Cells obtained from cervical lymph nodes were analyzed by flow cytometry. **(A)** Comparison of the number of single/live/CD45^+^CD3^+^CD4^+^ T lymphocytes between periodontal tissues and cervical lymph nodes. Flow cytometric quantification of **(B**, **C)** CD45^+^CD3^+^CD4^+^T-bet^+^ Th1 lymphocytes, **(D**, **E)** CD45^+^CD3^+^CD4^+^RORγt^+^ Th17 lymphocytes, and **(F**, **G)** CD45^+^CD3^+^CD4^+^AhR^+^ Th22 lymphocytes in periodontal lesions upon infection with the *A. actinomycetemcomitans* VT1169, *rmlC*, or *rmlC/rmlC^+^* strains, or the untreated (UT) controls. Data were pooled from three independent experiments (n=3). Mean ± SD, one-way ANOVA and Holm-Sidak post-hoc test, *p < 0.05. Error bars represent SEM in all panels.

We next assessed whether the variations observed in the periodontal lesions are reflected in the frequency and absolute number of Th1, Th17, and Th22 lymphocytes within the oral-draining lymph nodes. We observed discrepancies in the absolute number of total T helper cells between the periodontal lesions and cervical lymph nodes (**Figure 4A**). To further determine whether the specific T helper subsets showed similar variations to those found in periodontal lesions, the frequency and absolute number of T-bet^+^, RORγt^+^, and AhR^+^ T lymphocytes were determined in the cervical lymph nodes. Similarly, neither the frequency nor the absolute cell number of T-bet^+^, RORγt^+^, or AhR^+^ T lymphocytes significantly varied among the different experimental conditions (**Figures 4B–G**). Therefore, the decreased Th1, Th17, and Th22 lymphocyte detection in the periodontal lesions induced with the *A. actinomycetemcomitans* *rmlC* mutant strain was not reflected in changes in T helper lymphocyte detection in the cervical lymph nodes that drain these periodontal lesions.

### The O-PS-Defective *A. actinomycetemcomitans* Serotype *b* Mutant Strain Induces Less Expression of CD40 and CD80 in Dendritic Cells and B Lymphocytes Than the Wild-Type Strain

**Figure 5:**
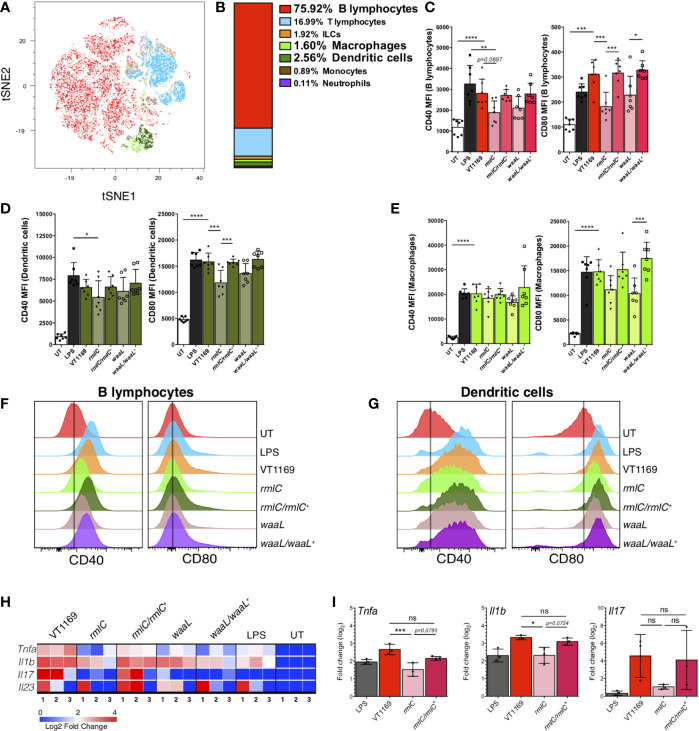
B-lymphocyte, dendritic cell, and splenocyte responses against the different strains of *A. actinomycetemcomitans*. **(A)** tSNE map of 11-parameter flow cytometry data from untreated (UT) mice, color-coded as follows: B-cells in red, T-cells in cyan, innate lymphoid cells (ILCs) in orange, macrophages in light green, dendritic cells in dark green, monocytes in brown, and neutrophils in purple. **(B)** Percentage of each cell population described in **(A)**. Data pooled from two independent experiments (n=4). The MFI levels of the co-stimulatory molecules CD40 and CD80 are shown in **(C)** for B lymphocytes, **(D)** for dendritic cells, and **(E)** macrophages stimulated 20 h with the *A. actinomycetemcomitans* VT1169, *rmlC*, *rmlC/rmlC^+^*, *waaL*, or *waaL/waaL^+^* strains, and the *E. coli*-derived LPS-stimulated and UT cells, used as controls. The histograms representing the MFI levels of the co-stimulatory molecules CD40 and CD80 are shown in **(F)** for B lymphocytes and **(G)** for dendritic cells, under the same conditions experimental described in **(C-E)**. Data pooled from three different experiments (n=7). **(H)** Heatmap and **(I)** bar plots of qRT-PCR analysis of *Tnfa*, *Il1b*, *Il17*, and *Il23* transcripts in total splenocytes stimulated 8 h with the *A. actinomycetemcomitans* VT1169, *rmlC*, *rmlC/rmlC^+^*, *waaL*, or *waaL/waaL^+^* strains, and the *E. coli*-derived LPS-stimulated and UT cells, used as controls (n=3). Data pooled from two independent experiments. Mean ± SD, one-way ANOVA and Tukey post-hoc test, *p < 0.05, **p < 0.01, ***p < 0.001, ****p < 0.0001. Error bars represent SEM in all panels. *Il*, interleukin; LPS, *E. coli*-derived lipopolysaccharide; *Tlr*, toll-like receptor; *Tnfa*, tumor necrosis factor-alpha.

Since T helper cell polarization and expansion requires not only the presence of bacterial antigen and cytokines, but also co-stimulatory molecules expressed by pathogen-experienced antigen-presenting cells (APCs), including dendritic cells (DCs), macrophages, and B lymphocytes (35), we examined the surface expression of the co-stimulatory molecules CD40 and CD80 in mice APCs. First, to determine the frequency of APCs present in the mice periodontal tissues at steady state, a flow cytometry characterization of the immune cell network followed by a dimensionality reduction with the T-distributed stochastic neighbor embedding (tSNE) algorithm was carried out (**Figure 5A**). Using a panel that allowed us to detect B lymphocytes (CD45^+^CD19^+^MHC-II^hi^), T lymphocytes (CD45^+^CD3^+^CD90^+^), innate lymphoid cells (CD45^+^CD90^+^CD3^-^), macrophages (CD45^+^CD64^+^CD11b^+^), DCs (CD45^+^CD11c^+^MHC-II^hi^), neutrophils (CD45^+^Ly6G^+^Ly6C^mid^), and monocytes (CD45^+^Ly6C^+^Ly6G^-^) (**Supplementary Figure S4**), we determined that the main APCs present in the periodontal tissues were B lymphocytes (75.0%), DCs (2.56%), and macrophages (1.6%) (**Figure 5B**). These results were crucial to design a proper experimental set up to analyze the changes in APCs in periodontal lesions, while trying to emulate, as much as possible, the mice gingival barrier context *in vitro*.

Among the co-stimulatory molecules, CD40 and CD80 are critical signals expressed by APCs for optimal activation of T lymphocytes (36). Since we determined that B lymphocytes, DCs, and macrophages are the main APCs present in the mice periodontal tissues, we evaluated whether the lack of O-PS in the *A. actinomycetemcomitans* serotype *b* strain alters the CD40 and CD80 surface expression on bacteria-challenged splenocytes-derived APCs. Splenocytes were used based on the similarities in APC proportions between the spleen and periodontal tissues in mice (37) (**Supplementary Figure S5**). When B lymphocytes were challenged with the VT1169 wild-type strain, the expression levels of CD40 and CD80 augmented significantly, reaching similar levels to those expressed in B lymphocytes stimulated with *E*. *coli*-derived LPS, used as positive control (**Figures 5C, F**). The exposure of B lymphocytes to the *rmlC* or *waaL* mutant strains led to lower CD40 and CD80 expression levels to those detected in the VT1169 wild-type strain-challenged cells (**Figures 5C, F**). When the B lymphocytes were challenged with the *rmlC/rmlC^+^* or *waaL/waaL^+^* complemented strains, the expression levels of CD40 and CD80 augmented, reaching similar levels to those detected in B lymphocytes challenged with the VT1169 wild-type strain (**Figures 5C, F**). Similarly, the VT1169 wild-type strain-challenged DCs significantly increased the expression of CD40 and CD80, reaching similar levels to those expressed in DC stimulated with *E*. *coli*-derived LPS (**Figures 5D, G**). When the DCs were challenged with the *rmlC* mutant strain, only the expression of CD80 was significantly decreased as compared with VT1169 wild-type strain-challenged DCs (**Figures 5D, G**). In the DCs stimulated with the *waaL* mutant strain, the expression of CD80 was lower as compared with the DCs stimulated with the VT1169 wild-type strain; however, these differences were not statistically significant (**Figures 5D, G**). The stimulation of DCs with the *rmlC/rmlC^+^* or *waaL/waaL^+^* complemented strains significantly increased the expression of CD80, reaching similar levels to those detected in VT1169 wild-type strain-challenged DCs (**Figure 5D**). Although macrophages increased the surface expression of CD40 and CD80 after *E*. *coli*-derived LPS or *A. actinomycetemcomitans* strains exposure, no significant differences on CD40 and CD80 expression were found among the different bacteria-stimulated conditions (**Figure 5E**). Importantly, no significant differences in cell viability were detected in splenocytes challenged with the aforementioned strains (**Supplementary Figure S6**).

### The O-PS-Defective *A. actinomycetemcomitans* Serotype *b* Mutant Strain Induces Less Expression of *Tnfa* and *Il1b* in Splenocytes Than the Wild-Type Strain

Besides, the expression levels of the NF-κB target genes *Tnfa* and *Il1b*, as well as *Il17 and Il23*, were measured in total splenocytes stimulated with the aforementioned *A. actinomycetemcomitans* strains after 8 h, showing that the absence of the O-PS domain impaired the transcript levels of those proinflammatory cytokines (**Figure 5H**). In particular, when splenocytes were exposed to the *rmlC* mutant strain, the expression levels of *Tnfa*, *Il1b*, and *Il17* were lower to those detected on the VT1169 wild-type strain-challenged splenocytes; however, the compared levels of *Il17* showed no statistically significant differences (**Figure 5I**). When the splenocytes were challenged with the *rmlC/rmlC^+^* complemented strain, the expression levels of *Tnfa*, *Il1b*, and *Il17* augmented, reaching similar levels to those detected in splenocytes challenged with the VT1169 wild-type strain (**Figure 5I**). Collectively, these results demonstrate that the *A. actinomycetemcomitans* serotype *b* O-PS domain is key to trigger the gene expression of the proinflammatory cytokines *Tnfa* and *Il1b* on immune cells, and it is highly involved in the upregulation of the surface co-stimulatory molecules CD40 and CD80 on B lymphocytes and DCs.

## Discussion

*A. actinomycetemcomitans* strains belonging to the serotype *b* show higher *in vitro* and *in vivo* immunogenicity and are more frequently isolated from the periodontal lesions of periodontitis-affected patients when compared with the other serotypes. Within this serotype *b* cluster, most studies have focused on the pathogenic mechanisms associated with the leukotoxin, while the virulence and immunogenic potential of the conserved O-PS component of its LPS have been largely understudied. In the current study, we investigated the virulence and immunogenicity of a transposon-derived *A. actinomycetemcomitans* serotype *b* mutant strain, characterized by the deletion of the *rmlC* gene and the impaired expression of its O-PS. This O-PS-defective *A. actinomycetemcomitans* strain provoked lower alveolar bone resorption, decreased infiltration of Th1, Th17, and Th22 lymphocytes, and downregulated expression of *Ahr*, *Il1b*, *Il17*, *Il23, Tlr4*, and RANKL (*Tnfsf11*) in experimental periodontal lesions, as compared with the wild-type strain. These variations were focalized in the periodontal tissues, without significant disturbance in the T helper subsets within the cervical lymph nodes. During the characterization of the gingival immune compartment, we also determined that the most frequent periodontal APCs were B lymphocytes and DCs. In these cells, O-PS-defective *A. actinomycetemcomitans* strain challenge triggered a lower expression of the co-stimulatory molecules CD40 and CD80, as well as lower expression of *Tnfa* and *Il1b*, as compared with the wild-type strain, suggesting that the lack of this bacterial moiety could affect their capacity to prime T lymphocytes towards the pro-osteolytic phenotypes.

Consistent with previous reports (18, 32, 38, 39), our results demonstrated that the oral inoculation of the serotype *b* VT1169 wild-type strain was capable of inducing bone loss. Conversely, when the periodontal lesions were induced with the *rmlC* mutant strain lacking the O-PS moiety, the mice developed less severe alveolar bone loss. Based on this observation, we propose that the O-PS domain exerts a critical role in the virulence of the serotype *b* cluster. This may be due to changes in the periodontal immune cell network mediating the activation of tooth-supporting bone resorption in response to structural variations in bacterial LPS, as it was previously reported with the purified LPS of *A. actinomycetemcomitans* serotype *b* (40–42).

The immunodominant epitope of the LPS from the *A. actinomycetemcomitans* serotype *b* resides in its O-PS domain (33, 34). We have previously reported that serotype *b* induces higher alveolar bone resorption than the other serotypes, and this increased bone loss was associated with the Th1 and Th17-lymphocyte activity in periodontal lesions (18). Besides, the serotype *b* has demonstrated increased immunogenicity *in vitro*, as it exerts a key role in phagocytosis resistance by human neutrophils (43), promotes the formation of osteoclast-like cells from bone marrow cells (44), induces the release of monocyte and T lymphocyte chemotactic factors, such as CCL2 and IL-8, and the secretion of IL-1 in challenged murine macrophages (45), triggers the expression of proinflammatory cytokines in stimulated monocytes (46) and dendritic cells (21), and induces the production of RANKL in T lymphocytes (47). All of these capacities of *A. actinomycetemcomitans* serotype *b* have been attributed to the O-PS structure of its LPS. In the present study, when periodontal lesions were induced with *A. actinomycetemcomitans* lacking the O-PS domain, we observed a significant reduction in the transcript levels of the cytokines IL-1β, IL-17, and IL-23, the Th22-associated transcription factor AhR, and the osteoclastogenic mediator RANKL. In line with the data obtained from the *in vivo* experiments, splenocytes challenged with the *rmlC* mutant strain expressed significantly less transcript levels of TNFα and IL1β, ratifying the importance of the O-PS fraction in *A. actinomycetemcomitans* immunogenicity.

The decreased transcript levels of IL-1β induced by the *rmlC* mutant could be involved in its diminished virulence, as previous works have shown that IL-1β inhibition reduced bone loss in experimental periodontitis (48, 49). Nonetheless, alveolar bone resorption during periodontitis is mainly mediated by the Th17-type of immune response, which is central in the periodontal osteo-immune network crosstalk (50). In infected mice, the absence of O-PS also diminished the expression levels of the Th17-related cytokines IL-23 and IL-17. IL-23 is directly involved in the Th17 osteolytic phenotype maintenance and Th22 lymphocyte differentiation and could be secreted by APCs, contributing to the polarization of *naïve* T cells and the perpetuity of lymphocytic pathogenic activity. Further, IL-17, the signature Th17-type cytokine, is precisely involved in immune-mediated alveolar bone resorption by directly promoting osteoclastogenesis and inducing RANKL expression on periodontal osteoblasts and fibroblasts (51). In this context, we also found that the inoculation of the *rmlC* mutant strain ameliorated the expression of RANKL in periodontal lesions. Potentially, this perturbation in the cytokine milieu could be due to changes in a plethora of cells that have the ability to recognize the O-PS, including immune and tissue-resident cells. However, it is well known that the key players in the response against immunostimulatory oral pathogens mostly belong to the immune cell compartment. Herein, based on the significant perturbations observed in the periodontal Th1, Th17, and Th22-associated proinflammatory and pro-osteolytic cytokine milieu in the periodontal tissues, we can conclude that the O-PS from the *A. actinomycetemcomitans* serotype *b* is key in the occurrence of inflammatory events that lead to alveolar bone destruction. Whether these perturbations are mainly mediated by immune cells is a matter of future research.

To dissect the influence of the O-PS in the gingival immune cell compartment, we analyzed the changes in the frequency and absolute number of T helper subsets in the periodontal tissues, focusing on the Th1 and Th17 proinflammatory and osteoclastogenic subsets. Our previous works have revealed that *A. actinomycetemcomitans* serotype *b* induces greater infiltration of Th1 and Th17 cells within experimental periodontal lesions, as compared with serotypes *a* or *c* (18). Besides, the serotype *b-*induced alveolar bone loss was also associated with increased infiltration of IL22^+^AhR^+^ Th22 lymphocytes within periodontal lesions (32). Indeed, IL-22 levels correlated with RANKL expression in periodontal lesions, suggesting a role of Th22 lymphocytes in the RANKL-mediated bone loss (32). In the present study, we found that the lack of O-PS in the *rmlC* serotype *b* mutant strain caused less infiltration of Th1, Th17, and Th22 lymphocytes in periodontal lesions as compared with the wild-type strain. In this context, Th1 lymphocytes are the main producers of the proinflammatory cytokines IL-1β, IL-12, IFN-γ, and TNF-α, with a transcriptional program controlled by the transcription factor T-bet. In fact, *A. actinomycetemcomitans*-induced periodontitis in IFN-γ-knockout mice resulted in decreased alveolar bone resorption accompanied by an impaired host defense against microbial dissemination followed by mice death, showing the key role of the Th1-related type-I IFN signaling in the local immune-mediated pathological bone loss and host protective response against *A. actinomycetemcomitans* (52). Thus, the diminished bone loss observed in our experimental approach could be in part due to impaired Th1 differentiation and/or infiltration into periodontal lesions. Accordingly, the ablation of CCL3/CCR1/CCR5 Th1-associated chemotactic axis in mice challenged with *A. actinomycetemcomitans* protected them from alveolar bone loss due to decreased Th1 lymphocyte infiltration (53). Even so, Th17 lymphocytes are considered the main bridge between immune response and bone metabolism due to their ability to directly express RANKL and induce RANKL expression in periodontal fibroblasts through IL-17 production (51). In turn, Th22 lymphocytes have also been described as an osteoclastogenic T helper subset (54). Similar to IL-17, IL-22 can directly promote osteoclast differentiation (54) and induce RANKL expression in periodontal ligament fibroblasts (55). Our results showed that components of the periodontal immune cell compartment are able to detect changes in the structure of O-PS from the *A. actinomycetemcomitans* LPS, which resulted in the modification of the local immune response mediated by the Th1, Th17, and Th22 lymphocytes. We cannot totally discard that other immune players could exert a role in mediating alveolar bone loss in response to variations in the presence of O-PS, including other T lymphocyte subsets such as Th2, T regulatory, or γδ-T cells, among others. Despite the fact that, to date, the knowledge of the oral immune landscape is still limited, most of the studies point to the protagonism of these three T helper subsets, particularly to the Th1 and Th17 lymphocytes, during the immuno-mediated alveolar bone resorption. In this regard, future studies are needed to dissect in deep all the mechanisms and immune cells participating in the variability of bone loss observed when the O-PS moiety of *A. actinomycetemcomitans* was absent.

Interestingly, the variation pattern observed at the periodontal level was not mirrored at the cervical lymph nodes, as could be expected. Whether these differences are an effect of variations in the leukocyte trafficking, tissular disturbances in chemotactic signals, or due to local changes in the orchestration of T helper cell differentiation at tertiary lymphoid structures is something that, unfortunately, was not addressed in this study and it needs to be further investigated. Overall, together with the fact that the *rmlC* mutant strain causes a reduced proinflammatory and osteoclastogenic cytokine network in the periodontal lesions, our data suggest that the lack of O-PS domain may interfere with the local mechanisms of inflammation and alveolar bone resorption mediated by Th1, Th17, and Th22 lymphocytes. These results, however, should be interpreted with caution, as the approach used here to detect T helper subsets was based only on their master transcription factors. It is known that Foxp3^+^ regulatory T cells could co-express RORγt and maintain or even enhance their suppressive capacity (56). Based on that, we cannot rule out the possibility that RORγt^+^ T cells are also displaying regulatory functions in our system. To solve this, future studies dissecting the heterogeneity and functional plasticity of the analyzed T helper subsets in our model of experimental periodontitis are needed.

The activation of the adaptive immunity depends on co-stimulatory surface molecules expressed by APCs that regulate the magnitude and quality of the T helper cell response, including CD40, CD80, and CD86. Indeed, the aforementioned Th1, Th17, and Th22 lymphocytes require these signals to orchestrate an appropriate adaptive immune response. The recognition of peptide/MHC II complexes on DCs by CD4^+^ T lymphocytes upregulates the CD40 ligand (CD40L), and the CD40/CD40L interactions, in turn, ‘license’ DCs for T-cell priming by overexpressing CD80 and CD86 (57). We found that the absence of O-PS affected the surface expression of the co-stimulatory molecules CD40 and CD80 in B lymphocytes and DCs, thus potentially disturbing the priming capacity of these APCs. This could explain, at least in part, the variation in frequency and number of the different T helper subsets that we detected in the periodontal tissues. Additionally, our results revealed that DCs exert higher surface expression of CD40 and CD80, as compared with B lymphocytes, emphasizing their role as professional APCs. Similar results were found when human DCs were exposed to mutant variants of *Neisseria meningitidis* LPS lacking O-PS, which exhibited a decreased surface expression of CD80 and CD86 (58). Otherwise, DCs that lack CD40 and CD80 have enhanced ability to induce T regulatory cells, while weakly direct other T helper cell subsets differentiation (59). Furthermore, the higher expression of CD40 on licensed DCs may favor the Th1 differentiation (60). Based on our results, we could establish that APCs, and particularly DCs and B lymphocytes, may be able to sense the presence of O-PS in the *A. actinomycetemcomitans* LPS structure, affecting their priming capacity and the quality of the T-cell mediated immune response. Further studies are needed with a focus on the pattern recognition receptor (PRR) signaling to determine the pathways involved in the recognition of O-PS by periodontal APCs. In this context, the immune response against *P. gingivalis* LPS is unusual due to its unique and heterogenous lipid A structure, being considered an agonist for TLR-2 as well as an agonist or antagonist for TLR-4 (61, 62). In fact, *P. gingivalis* LPS or its purified lipid A moiety were able to activate TLR4-deficient macrophages obtained from C3H/HeJ mice, demonstrating their signaling *via* TLR-2 (63, 64). However, the lipid A moiety is highly conserved in *A. actinomycetemcomitans* and taxonomically close species, such as *Aggregatibacter aphrophilus* (65, 66), highlighting the potential role of the O-PS in the variability of *A. actinomycetemcomitans* immunogenicity. Thus, the understanding of the variability in the *A. actinomycetemcomitans* O-PS/PRR signaling could provide evidence to further elucidate potential mechanisms underlying our findings.

The *rmlC* gene has also been involved in the mediation of leukotoxin secretion *via* the TolC-dependent type I secretion system (T1SS) (27), and in the collagen adhesion, through glycosylation of the extracellular matrix protein adhesin A (EmaA) (26). Hence, we cannot rule out that our results are, at least in part, also associated with changes in these virulence mechanisms potentially affected by the deletion of the *rmlC* gene in *A. actinomycetemcomitans*. However, the leukotoxin is an exotoxin associated with immune evasion through the induction of lymphocytes apoptosis (67); thus, increased T-cell survival and consequently, enhanced local T-cell mediated immune response would be expected with a reduction in the leukotoxin production, instead of the immune response ablation observed in our findings. Besides, no differences were found in periodontal T helper cell survival when challenged with the wild-type, mutant, and complemented strains of *A. actinomycetemcomitans* (**Supplementary Figure S3**). Since leukotoxin is a primate-specific exotoxin (23) and we used a murine model of periodontitis and mouse-derived splenocytes, we could speculate that the effects on leukotoxin secretion related to the *rmlC* gene must be exiguous in the immune perturbations observed in our experimental approaches. On the other hand, since the periodontal lesions were induced by direct bacterial microinjections into periodontal tissues, avoiding interactions with commensal microbiota and gingival keratinocytes, the role of adhesins needed for bacterial colonization should not be playing a major role in the observed periodontal immune changes.

In the current study, *A. actinomycetemcomitans* strains belonging to the serotype *b* were used in all experiments due to their higher immunogenic potential as compared with the other serotypes. Indeed, *A. actinomycetemcomitans* strains belonging to the serotype *b* have demonstrated a higher capacity to trigger *Ifng*, *Tnfa*, *Il1b*, *Il6*, *Il12*, and *Il23* expression in human monocyte-derived DCs as compared with *A. actinomycetemcomitans* strains belonging to the other serotypes (21). Besides, different *in vitro* and *in vivo* studies have ratified the higher immunogenicity and virulence of serotype *b* ATCC 43718 strain as compared with serotypes *a* or *c* (18–21, 29, 47). Interestingly, the *A. actinomycetemcomitans* strains ATCC 29522, ATCC 43718, and ATCC 29524, all of them belonging to serotype *b*, induced similar overexpression of *Ifng*, *Tnfa*, *Il1b*, *Il6*, *Il12*, and *Il23*, demonstrating that the higher immunogenic potential attributed to the serotype *b* is conserved (21). Even so, considering the genetic diversity of *A. actinomycetemcomitans*, more studies are needed to ratify the role of the O-PS from *A. actinomycetemcomitans* serotype *b*, for example, by using different strains belonging to this serotype. Overall, our findings contribute to a better understanding of a virulence factor that could be involved in the higher immunogenicity and virulence of *A. actinomycetemcomitans* serotype *b* strains. Accordingly, previous *in vitro* data already suggested higher immunogenicity of serotype *b* O-PS as compared with serotypes *a* or *c* O-PS. Indeed, murine macrophages challenged with O-PS purified from *s*erotype *b* produced higher levels of IL-1 than the same cells challenged with O-PS purified serotypes *a* or *c* strains (45). Despite this, to gain a deep understanding of how the O-PS from serotypes *a*, *b*, and *c* contribute to the distinct immunogenicity and virulence of *A. actinomycetemcomitans*, future studies addressing their effects *in vivo* are needed, ideally using clinical isolates.

From a translational view, our findings contribute to a better understanding of the virulence factors involved in the immunostimulatory and pathogenic potential of *A. actinomycetemcomitans*. In particular, herein, we demonstrated the role of *A. actinomycetemcomitans* O-PS in the triggering of critical pathological mechanisms involved in the clinical presentation of periodontitis. Several epidemiological studies have clearly reported the direct association between *A. actinomycetemcomitans* strains belonging to the serotype *b* and severe forms of periodontitis (2, 15–17). Thus, the gained insights on the virulence mechanisms of this group of strains could help to increase the accuracy of diagnosis, treatment, and prognosis of periodontitis. Similarly, the better comprehension of the pathogenic mechanisms of *A. actinomycetemcomitans* involved in the pathogenesis of other systemic conditions, including rheumatoid arthritis (68) and atherosclerosis (69), could contribute to understanding how oral conditions could affect the systemic health, particularly due to the systemic dissemination of oral bacteria, such as *A. actinomycetemcomitans*. In this regard, special emphasis should be put on the development of precise microbiological diagnostic tools that could allow identifying the presence of highly virulent *A. actinomycetemcomitans* strains, thus better-determining risk profiles and personalized treatments of oral and oral-related systemic diseases.

Recent epidemiological studies have revealed that the prevalence of periodontitis is higher in males than females; thus, potential sex-related periodontitis susceptibility needs to be considered (70). Indeed, some evidence points to sex dimorphism related to sex steroids effects on the host’s immune responses and the control of subgingival microbiota, being estrogen associated with a decreased susceptibility to periodontitis (71, 72). However, mice models of periodontitis have revealed an opposite trend, with higher susceptibility to alveolar bone loss and periodontal inflammation in female mice as compared with male mice (73). In the present study, only female mice were used, due to the higher increased periodontitis susceptibility could allow detecting small differences between the mice periodontal status. Nonetheless, more studies are needed to elucidate the potential role of sex-specific determinants on the capacity of *A. actinomycetemcomitans* O-PS to induce periodontitis.

To our knowledge, this is the first study reporting the role of O-Polysaccharide in the immunogenicity and virulence of *A. actinomycetemcomitans* serotype *b* in an *in vivo* model of periodontitis. Our results confirm that this bacterial moiety affects the maturation of APCs, the Th1, Th17, and Th22-pattern of the periodontal immune response, and the alveolar bone resorption during experimental periodontitis, playing a significant role in the virulence and immunogenicity of this highly virulent periodontal bacteria.

## Data Availability Statement

The raw data supporting the conclusions of this article will be made available by the authors, without undue reservation.

## Ethics Statement

The animal study was reviewed and approved by Institutional Committee for Animal Care and Use from Universidad de Chile (Ethical permit #061601) and the Stockholm Regional Ethics Committee.

## Author Contributions

GM conceived the study, designed and performed most experiments, analyzed the data, and was involved in drafting the article. FC, EC, JA, and CT-A performed experiments. AH and EV designed experiments, analyzed the data, and critically evaluated and supplemented the article. RV conceived the study, designed experiments, analyzed the data, and prepared the article for submission. All authors contributed to the article and approved the submitted version.

## Funding

This study was financially supported by grant FONDECYT 1181780 from the Chilean Governmental, Agencia Nacional de Investigación y Desarrollo (ANID). GM is a recipient of a Ph.D. Scholarship CONICYT 21170297 from ANID.

## Conflict of Interest

The authors declare that the research was conducted in the absence of any commercial or financial relationships that could be construed as a potential conflict of interest.
