# Supplementary material for: O-Polysaccharide Plays a Major Role on the Virulence and Immunostimulatory Potential of Aggregatibacter actinomycetemcomitans During Periodontal Infection
Source: Front Immunol. 2020 Oct 30;11:591240. doi: 10.3389/fimmu.2020.591240 (PMC7662473; doi:10.3389/fimmu.2020.591240)
Supplement: Supplementary file 1 [file DataSheet_1.pdf]

Supplementary Figure S1

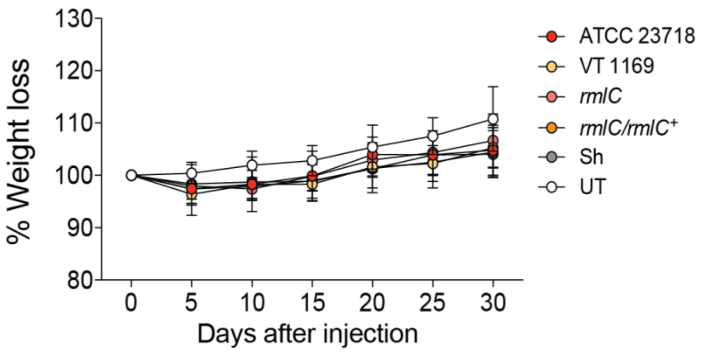

SUPPLEMENTARY FIGURE S1. Percentage of body-weight loss in periodontally infected mice. Measurements of body-weight loss in mice infected with *A. actinomycetemcomitans* ATCC 23718, VT1169, *rmlC*, or *rmlC/rmlC*$^{+}$ strains, and sham-infected (Sh) and untreated (UT) controls. 100% is considered as the body-weight of each mouse at the initial time-point (n=4).

Supplementary Figure S2

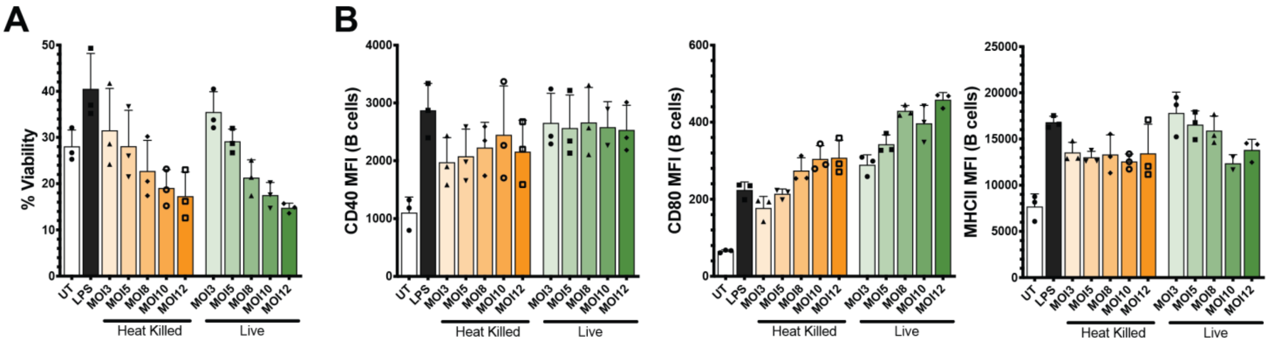

SUPPLEMENTARY FIGURE S2. Dose-response *in vitro* assays of splenocytes stimulated with increasing multiplicity of infection of heat-killed or live *A. actinomycetemcomitans* VT1169 strain. (A) Percentage of live splenocytes stimulated at different multiplicity of infections **(**MOI=3, MOI=5, MOI=8, MOI=10, or MOI=12) of heat-killed or live *A. actinomycetemcomitans* VT1169 strain for 20 h (n=3). Splenocytes stimulated with *E.coli*-derived LPS (LPS) and untreated (UT) cells were used as controls (n=3). **(B)** Mean fluorescence intensity (MFI) levels of the co-stimulatory molecules CD40, CD80, and MHCII from the same conditions described in **A**. The data were pooled from two independent experiments. Error bars represent SEM in all panels.

Supplementary Figure S3

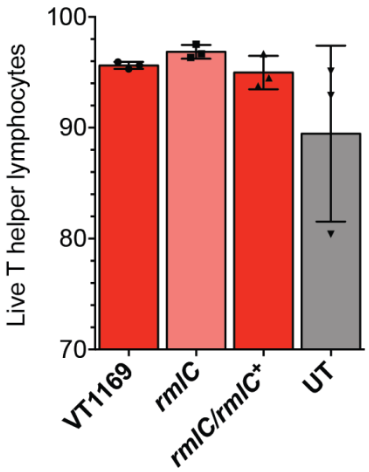

SUPPLEMENTARY FIGURE S3. T helper lymphocyte viability in the periodontal lesions induced with the *A. actinomycetemcomitans* VT1169, *rmlC*, or *rmlC/rmlC*$^{+}$ strains. Percentage of live T helper lymphocytes from the total CD45^{+}CD3^{+}CD4^{+} cells obtained from periodontal tissues of mice infected with the *A. actinomycetemcomitans* VT1169, *rmlC*, and *rmlC/rmlC*$^{+}$ strains, and untreated (UT) controls (n=3). The data were pooled from three independent experiments. Mean ± SD, one-way ANOVA, and Holm-Sidak post-hoc test. Error bars represent SEM.

Supplementary Figure S4

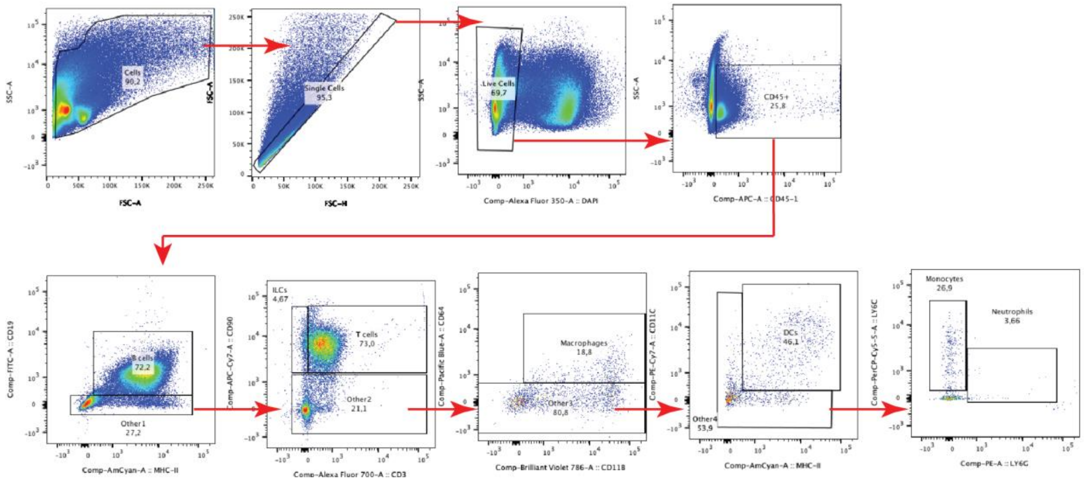

SUPPLEMENTARY FIGURE S4. Flow cytometry gating strategy used for periodontal tissues immune cell compartment characterization.

Supplementary Figure S5

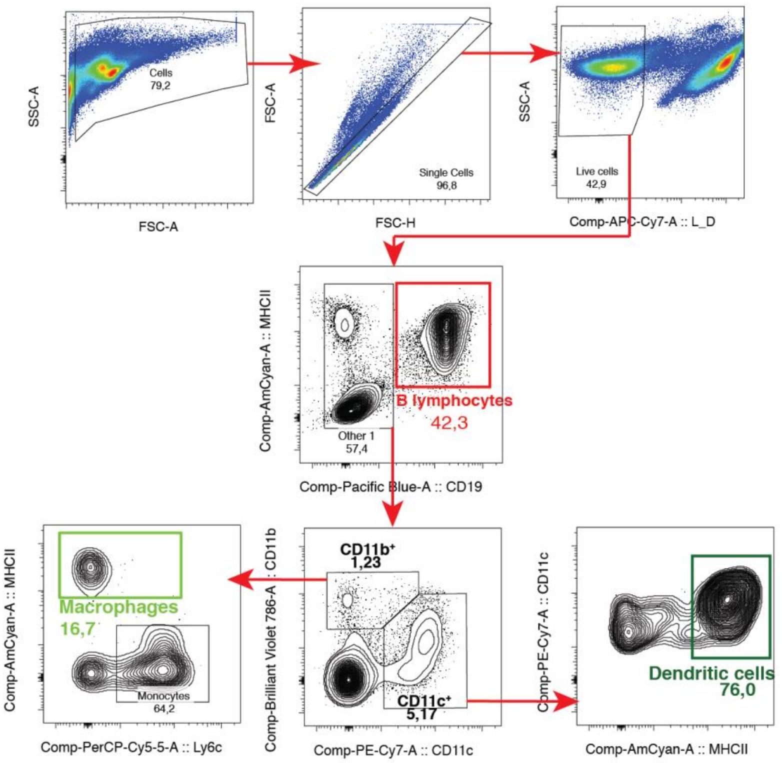

SUPPLEMENTARY FIGURE S5. Flow cytometry gating strategy used for the *in vitro* experiments with total splenocytes.

Supplementary Figure S6

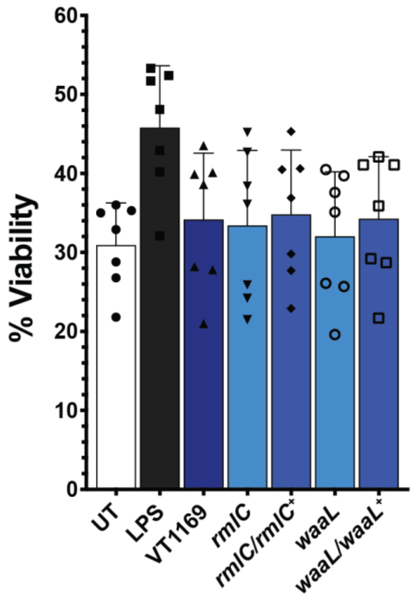

SUPPLEMENTARY FIGURE S6. Splenocytes viability upon challenge with the *A. actinomycetemcomitans* VT1169, *rmlC*, *rmlC/rmlC*$^{+}$, *waaL*, or *waaL/waaL*$^{+}$ strains. Percentage of live splenocytes stimulated at a multiplicity of infection MOI=3 with the *A. actinomycetemcomitans* VT1169, *rmlC*, *rmlC/rmlC*$^{+}$, *waaL*, or *waaL/waaL*$^{+}$ strains for 20 h (n=7). Splenocytes stimulated with *E. coli*-derived LPS (LPS) and untreated (UT) cells were used as controls (n=7). The data were pooled from three independent experiments. Mean ± SD, one-way ANOVA, and Tukey post-hoc test. Error bars represent SEM.

Supplementary Table S1

SUPPLEMENTARY TABLE S1 Forward and reverse primers used for amplifications by qRT-PCR.

**Primer**	**Forward**	**Reverse**
Aa 16S rDNA	cggaaatggaatgcttgc	ctgaggaagcctagcaat
Ahr	gccaagagcttctttgatgg	tgctgaaagcccaggtaatc
Hprt	tcagtcaacgggggacataaa	ggggctgtactgcttaaccag
Ifng	ggaggaactggcaaaaggat	ttcaagacttcaaagagtctgagg
Il-1b	agttgacggaccccaaaag	tttgaagctggatgctctcat
Il-23	tgttgccctgggtcactc	gagcccagtcaggactgcta
Il-6	tgatggatgctaccaaactgg	ttcatgtactccaggtagctatgg
Il-10	cagagccacatgctcctaga	tgtccagctggtcctttgtt
Il-17	cagggagagcttcatctgtgt	gctgagctttgagggatgat
Il-22	gtggagagatcaaggcgatt	cagacgcaagcatttctcag
Il-23	agcttcatgcctccctactg	ctgctgagtctcccagtggt
Tlr2	ggggcttcacttctctgctt	agcatcctctgagatttgacg
Tlr4	ggactctgatcatggcactg	ctgatccatgcattggtaggt
Tnfa	ctgtagcccacgtcgtagc	ttgagatccatgccgttg
RANKL (*Tnfsf11*)	tgaagacacactacctgactcctg	cccacaatgtgttgcagttc
18s	gcaattattccccatgaacg	gggacttaatcaacgcaagc

Aa, *Aggregatibacter actinomycetemcomitans*; *Ahr*, transcription factor aryl hydrocarbon receptor; *Hprt*, hypoxanthine phosphoribosyl transferase 1; *Ifng*, interferon-gamma; *Il*, interleukin; RANKL, receptor-activator of nuclear factor κB ligand; *Tlr*, toll-like receptor; *Tnfa*, tumor necrosis factor-alpha; *Tnfsf*, tumor necrosis factor super-family 11.
